# Extracellular Vesicles for The Diagnosis of Alzheimer's Disease and Related Dementias: A Diagnostic Accuracy Meta‐analysis

**DOI:** 10.1002/alz.093432

**Published:** 2025-01-09

**Authors:** Hash Brown Taha, Fatemah Yacoub, Alanna Morris, Allison Birnbaum

**Affiliations:** ^1^ USC Leonard Davis School of Gerontology, Los Angeles, CA USA; ^2^ University of California Irvine, Irvine, CA USA; ^3^ University of California, Los Angeles, Los Angeles, CA USA

## Abstract

**Background:**

Alzheimer’s disease, along with other cognitive disorders such as frontotemporal dementia (FTD), dementia with Lewy body (DLB) Parkinson’s disease dementia (PDD), and vascular cognitive impairment and dementia (VCD), comprise a range of conditions with similar cognitive symptoms but different pathophysiology. Currently, there is no biomarkers to distinguish them before death. The pathophysiological mechanisms differ significantly across these disorders, which implies a varied response to potential treatments. Furthermore, MCI often acts as an early phase, frequently leading to dementia, especially AD. Here, we assessed the diagnostic accuracy of biomarkers in general extracellular vesicles (EVs) and speculative CNS‐enriched EVs using the receiver operating characteristic (ROC) models to discriminate each disorder from one another or from healthy controls (HCs).

**Method:**

We conducted a PRISMA‐guided meta‐analysis on 49 studies to evaluate the diagnostic accuracy of biomarkers in general and speculative CNS‐enriched EVs for AD, FTD, DLB, PDD, VCD, MCI, and HCs, using hierarchical bivariate modeling of ROC statistics. Sub analyses were performed across EV types, mediums (e.g., CSF, plasma, serum, urine, saliva), isolation methods, among others. We also assessed publication bias and the potential number of missing studies with the trim‐and‐fill method and other robust statistical methods.

**Results:**

The analyses reveal that biomarkers derived from general EVs exhibit better diagnostic accuracy and less heterogeneity, variance, and publication bias than speculative CNS‐enriched EVs for all disease comparisons.

**Conclusion:**

These findings suggest that the usage of biomarkers derived from general EVs is superior to those from speculative CNS‐enriched EVs for the diagnosis of dementia.

